# The Potential of Human Monoclonal IgE Antibodies to Establish Biological Potency and Stability of Allergen Extracts

**DOI:** 10.1007/s11882-024-01168-4

**Published:** 2024-07-24

**Authors:** Ronald L. Rabin

**Affiliations:** Center for Biologics Evaluation and Research, US Food and Drug Administration, 10309 New Hampshire Avenue Building 52, Room 3332, Silver Spring, MD USA

**Keywords:** Allergy, Allergen extract, Allergen immunotherapy, Potency, standardization, ELISA, IgE, IgG4, IEMA

## Abstract

**Purpose of Review:**

Allergenic extracts are often standardized to control for potency, either by measuring concentrations of major allergens or “overall allergenicity” by competition for IgE in pooled sera from highly allergic subjects with a reference extract. Recent developments present an opportunity to use human mAb cloned from highly allergic subjects to define potency of allergenic extracts.

**Recent Findings:**

Two recent developments present an opportunity for revising potency measurements of allergen extracts: cloning allergen specific IgE from allergic subjects and extensive epitope mapping of major allergenic proteins.

**Summary:**

Because human IgE mAb recognize biologically relevant epitopes, they present a novel opportunity to determine the potencies of allergenic extracts and may contribute to the science base for allergen standardization.

## Introduction

Allergenic extracts are complex mixtures of allergenic and non-allergenic proteins used to aid in the diagnosis of, or to treat allergic disease. Since the advent of allergen immunotherapy over a century ago, the procedure of extracting proteins from pollen or animal hair is essentially unchanged. What has changed is the relatively recent advent of standardizing extracts by establishing quantitative measures potency. In the US, the potency measurements are compared to a reference extract chosen and distributed by the FDA’s Center for Biologics Evaluation and Research (CBER), which allows for comparison of extracts between manufacturers [1]. In the EU, potency measures are compared to the manufacturers’ in-house reference.

In September 2023, the Allergen Standardization Subcommittee met at the 16th International Paul-Ehrlich Seminar meeting in Frankfurt, Germany. Subcommittee members agreed that human IgE mAb present an opportunity to standardize allergen extracts with reagents that are inherently linked to the mechanism of allergic disease. Here I discuss allergen standardization and the potential advantages of applying IgE mAb towards standardization of allergen extracts.

## Allergen Standardization and Potency Measures in the United States (US) and European Union (EU)

In the US, potency values for allergen extracts may be defined by concentrations of their single major allergenic proteins, which among other characteristics, are proteins to which *≥* 50% of allergic patients react [2, 3]. For short ragweed pollen and cat hair and cat pelt extracts, which have a single major allergenic protein, the assay is radial immunodiffusion (RID), in which extract diffuses into agarose embedded with polyclonal specific sheep antisera to Amb a 1 or Fel d 1, respectively. Once fixed with acetic acid, a precipitin antigen-antibody ring forms, the diameter of which correlates with the antigen concentration in the extract. While the RID has served its purpose well, the assay can be cumbersome, sheep polyclonal antisera may recognize epitopes irrelevant to human allergic disease and may also vary from donor to donor, and there is no indication that the target antigen is intact.

For extracts that lack major allergens (or in which major allergens were not defined at time of standardization), potency values were determined by skin testing highly allergic subjects with 3-fold serial dilutions of extract [4]. These values were then transposed to surrogate immunoassays for lot release of manufactured extracts. Such is the case for standardized grass and house dust mite (HDM) extracts, for which the surrogate immunoassay is a competitive ELISA in which the test extract competes with a reference extract for specific IgE in sera pooled from 10 to 15 highly allergic donors. While these assays for quantitation of “overall allergenicity” are used in the US for grass and HDM extracts, and are the primary method of standardization in the EU, they are not ideal because of well-known limitations that include the labor and expense to replace sera pools, dependency on highly allergic donors, variability in the IgE specificity profile among serum pools, and that they provide no information about concentrations of any single allergen or isoforms [5]. In addition, since most atopic donors are multiallergic, their sera may contain IgE that recognize irrelevant allergens which can interfere with the assay if the extract contains cross-reactive allergens. For example, when screening sera for HDM extract, it is important to screen out sera from individuals who are sensitive to shellfish and have high levels of IgE against tropomyosin—a dominant shellfish allergen but a minor house dust mite allergen [6].

In the US, the US Food and Drug Administration’s Center for Biologics Evaluation and Research (CBER) determines which allergenic extracts are standardized. As stated in the Code of Federal Regulations, once potency measures have been determined, manufacturers must measure the allergenic activity of the product and state the potency on the label of each vial [7]. To facilitate compliance with the regulation, CBER maintains a reference reagent program to provide reference reagents to manufacturers for potency testing in which stocks are maintained and reagents are replaced when stocks are depleted. Rather than use CBER’s reference reagents, manufacturers may seek approval to use an alternative test method that provides an equally reliable measure of product potency and meet regulatory requirements. Regardless of the test, however, manufacturers must use the unitage of potency that CBER assigned to the product.

In the EU, extracts are standardized by manufacturers using “in-house” reference reagents that allow for lot-to-lot comparisons for each manufacturer’s products. However, because reference reagents are not shared across manufacturers and as discussed above, because sera pools used for potency measurements may vary, products across manufacturers cannot be compared.

## Allergen IgE Epitopes

Allergens are defined as molecules that induce and bind specific IgE antibodies. Since allergens activate mast cells or basophils by cross-linking two FcεR1 molecules, the allergen must either have two distinct epitopes or a repeating epitope that is properly spaced to bring the FcεR1 molecules in proximity. Theoretically any antigen can also be an allergen, but allergens are generally restricted to 30–40 protein families some of which activate the innate immune system through pattern recognition or protease-activated receptors, and others, such as seed storage proteins that are highly expressed in foods [2].

Identifying IgE epitopes is important to ensure that natural allergen extracts reflect patients’ sensitivity profiles, and for devising novel therapies, such as hypoallergens [8] and passive immunization with anti-allergen IgG mAbs [9]. IgE epitopes can either be linear—binding to a stretch of continuous amino acids, or conformational—binding to discontinuous peptide segments that is dependent on proper protein folding [10]. The concept that conformational epitopes are most common to environmental allergies while food allergens generally have linear epitopes may be due to the abundance and comparative ease of constructing peptide libraries [11], including the availability of commercial technologies such as Allerscan [12, 13]. Combining the technology of cloning human IgE mAb with cloning IgG4 blocking antibodies has led to identification of novel linear and conformational epitopes [14–18].

For Bet v 1, the most intensively studied environmental allergen, six conformational epitopes have been described [19–21], and even short peptide segments that have been described may have secondary structure, such as constrained loops or alpha helices that may require the protein to be structurally intact [19]. By contrast, there are multiple linear epitopes in the peanut allergens Ara h 2 and Ara h 6, some of which are considered “public” epitopes, those shared by a majority of peanut sensitive patients [13], and one of which repeats, which may contribute to the dominance of these proteins because a single IgE antibody is sufficient to cross-link IgE receptors and induce an allergic response [22]. However, there are also seven described conformational Ara h 2 IgE epitopes [19]; neutralization of two of them correlates with experimental protection against allergic responses and may correlate with desensitization to peanut after oral immunotherapy [23].

## Human IgE mAb: Linking Mechanism of Disease to Allergen Potency

A major mechanism of allergen immunotherapy is eliciting IgG4 blocking antibodies that compete with IgE for epitopes on the allergenic proteins, thereby blocking IgE binding and subsequent mast cell or basophil activation [24]. In that context, and to be consistent with in vivo skin reactivity assays, potency immunoassays should measure for intact protein and in particular IgE epitopes, be invariant over time, and in the case of extracts without single major allergenic proteins, define levels of the most important allergens. The recent development of IgE mAb cloned from allergic subjects satisfies these criteria. An additional advantage is that unlike mAb from laboratory animals, human IgE mAb have very high affinities as they are products of years of in vivo affinity maturation. Finally, properly stored, mAb are very stable reagents such that there is no concern that they will lose potency or specificity.

Currently, there are at least two laboratories who are cloning IgE mAb from allergic subjects. One, employed by IgGenix Inc., uses a fluorescence-activated cell sorting strategy to individually sort B cells, sequence the full-length heavy and light chains, and clone the IgE variable domains into human IgG4 expression vectors to express IgG4 mAb in Chinese hamster ovary cells [14]. Another, employed at Vanderbilt University, fuses IgE-secreting B cells to myelomas to create hybridomas that are single-cell sorted and expanded as clonal populations [25].

To test the utility of IgE mAb in measuring biological potency, CBER’s reference reagent laboratory, in collaboration with Robert Hamilton of Johns Hopkins University School of Medicine and Derek Croote and colleagues of IgGenix Inc. developed an ELISA using two IgE mAb cloned from B cells of cat-allergic donors and class switched to IgG_4_. We found that the new assay is equivalent to the current sheep polyclonal antibody-based RID in the measurement of potency of standardized cat allergen extracts [26].

As stated above, the RID is problematic, so while our immediate goal is to replace the Amb a 1 RID with an ELISA, cloning these novel mAbs (with or without class-switch) raises the possibility of expanding the repertoire of standardized extracts with antibodies that recognize established human IgE epitopes. These antibodies may not only facilitate standardization of extracts with single major allergens (e.g., birch and oak tree pollens), but also enable regulators to replace use of pooled sera with stable invariant reagents, thus enabling European regulators to achieve their goal of product comparability across manufacturers [5]. If so, assuming availability of reagents to regulators, what might be guiding principles towards selection of mAb clones?

- For allergen proteins with multiple IgE epitopes, choose mAb that recognize “public epitopes” shared by a sizable proportion of allergic individuals. Ideally, whether the epitopes are indeed public should be verified across a heterogenous set of patients, and for seasonal allergens, at different seasonal time points [27].
- If possible, consider cross-reactive epitopes that may allow for simultaneous standardization of a number of extracts. For example, the major allergen of pollens from trees of the order Fagales (birch, alder, hornbeam, hazel and white oak) are Bet v 1-like PR10 proteins [28]. Bet v 1 and Que a 1 are 58% identical and highly cross-reactive along with group 1 allergens from alder, hornbeam, hazel, chestnut and beech [28, 29], raising the possibility that a single pair of cross-reactive mAb can measure potencies of multiple extracts, (particularly if multiple species of oak can be included). In addition, mAb that recognize epitopes shared among peanut and tree nuts can potentially be used to measure potencies ofthose extracts [15].
- Preference towards conformational epitopes is ideal to confirm that allergens being measured are structurally intact in order to elicit IgG4 that directly competes for the IgE epitope.
- For allergen extracts without a single major allergenic protein that may be used to represent extract potency, consider a set of mAb to measure the most dominant proteins with the overall goal of quantifying the full set of allergens with a multiplexing technology such as LC/MS/MS [30]. For example, HDM extracts would have as potency measures levels of the three major proteins, groups 1, 2, and 23 allergens [2], supplemented with LC/MS/MS data for all 39 known allergens [31].
- While it may be attractive to use pools of mAb to replace pooled sera for measures of overall allergenicity, I suggest that the short-term gain of retaining a single representative number (e.g., BAU) is outweighed by defining the composition of extracts so that they may be matched to a patient’s profile derived from component-based testing [32] to move allergen immunotherapy towards personalized medicine, and motivate manufacturers towards developing more comprehensive extracts when warranted. While this goal may be best served with multiplexing technologies such as LC/MS/MS, it is likely that adoption of these methods will be “bridged” with immunological based tests, such as measurements of major allergens with human mAbs.

## Conclusion

The advent of human IgE mAb has the potential towards advancing allergen standardization by adding to the current number of extracts standardized in the United States and matching the advances in component resolved diagnostics.

## Data Availability

No datasets were generated or analysed during the current study.

## Abbreviations

AE: Allergen extract
CBER: US FDA Center for Biologics Evaluation and Research
EU: European Union
HDM: House dust mite
mAb: Monoclonal antibody
RID: Radial immunodiffusion assay
US: United States
